# Leptospirosis during Dengue Outbreak, Bangladesh

**DOI:** 10.3201/eid1105.041212

**Published:** 2005-05

**Authors:** Regina C. LaRocque, Robert F. Breiman, Mary D. Ari, Roger E. Morey, Firdous Ara Janan, John Mosely Hayes, M. Anowar Hossain, W. Abdullah Brooks, Paul N. Levett

**Affiliations:** * Massachusetts General Hospital, Boston, Massachusetts, USA;; † International Centre for Diarrhoeal Disease Research, Bangladesh, Dhaka, Bangladesh;; ‡ Centers for Disease Control and Prevention, Atlanta, Georgia, USA;; § Dhaka Medical College Hospital, Dhaka, Bangladesh;; ¶ Centers for Disease Control and Prevention, San Juan, Puerto Rico, USA

**Keywords:** Leptospirosis, Dengue, Bangladesh, fever

## Abstract

We collected acute-phase serum samples from febrile patients at 2 major hospitals in Dhaka, Bangladesh, during an outbreak of dengue fever in 2001. A total of 18% of dengue-negative patients tested positive for leptospirosis. The case-fatality rate among leptospirosis patients (5%) was higher than among dengue fever patients (1.2%).

Leptospirosis is a zoonotic infection caused by spirochetes of the genus *Leptospira*. Infection usually results when water or soil contaminated with the urine of an infected animal comes in contact with human skin or mucous membranes (1). Clinical manifestations of leptospirosis can range from a self-limited febrile syndrome to a fatal illness (Weil disease) characterized by hemorrhage, renal failure, and jaundice. In tropical settings, leptospirosis can be indistinguishable from other febrile illnesses such as scrub typhus, malaria, or dengue.

Although leptospirosis has been reported in neighboring areas of Southeast Asia (2,3), the disease is not recognized in Bangladesh, where diagnostic tests for leptospirosis are not available. However, environmental factors, such as floods, humidity, and water contamination, are amenable to spread of the disease in Bangladesh.

An epidemic of dengue fever and dengue hemorrhagic fever began in Bangladesh in 2000 (4), and a surveillance system was established to identify patients with denguelike illness at 2 major hospitals in Dhaka. Approximately three-quarters of patients meeting surveillance criteria had laboratory evidence of dengue infection. We hypothesized that leptospirosis might be a cause of illness among febrile patients who did not have dengue fever. To assess this, we retrospectively analyzed acute-phase serum samples from all dengue-negative patients by using real-time polymerase chain reaction (PCR) for *Leptospira*. We used data collected as part of the surveillance program to identify distinguishing clinical characteristics of leptospirosis.

## The Study

In 2000, the International Centre for Diarrhoeal Disease Research, Bangladesh (ICDDR,B) worked with staff from Dhaka Medical College and Holy Family Red Crescent Hospital to initiate surveillance for dengue as part of an emergency response to an epidemic of dengue and dengue hemorrhagic fever. Physicians at Dhaka Medical College and Holy Family Red Crescent Hospital were trained in the clinical diagnosis and management of dengue and dengue hemorrhagic fever according to World Health Organization guidelines. Patients hospitalized with fever and in whom a physician suspected dengue were enrolled in the surveillance program. Clinical and epidemiologic information, as well as acute-phase serum specimens, were systematically collected from surveillance patients. Acute-phase serum specimens were assessed for dengue virus antibodies by using a commercial immunoglobulin (Ig)G and IgM capture enzyme-linked immunosorbent assay (ELISA) (PanBio Dengue Duo, PanBio Ltd., Brisbane, Queensland, Australia) (5). In addition, serum samples collected from a subset of patients during the first 5 days of illness were evaluated for dengue virus RNA by using reverse transcriptase–polymerase chain reaction (RT-PCR), as described previously (6). Serum samples from patients with no dengue infection shown by antibody or RT-PCR testing were retrospectively assessed for leptospirosis by using a real-time PCR that amplifies the LipL32 gene (7), a virulence factor that is conserved among pathogenic *Leptospira* strains (8). Microplate *Leptospira* IgM ELISA testing (PanBio Ltd.) was conducted on all *Leptospira* PCR-positive serum specimens of sufficient quantity (9).

Specimens from 1,297 patients hospitalized at Dhaka Medical College and Holy Family Red Crescent Hospital between January 1 and December 31, 2001, were evaluated for dengue infection by using capture ELISA; 55 acute-phase serum samples were additionally evaluated by using RT-PCR for dengue. A total of 938 (72%) patients were diagnosed with dengue fever by serologic tests (932 patients0, RT-PCR (3 patients), or both (3 patients). Acute-phase serum specimens from the 359 patients without laboratory evidence of dengue were evaluated for leptospirosis; 63 (18%) had *Leptospira* detected by using PCR. Sixty-one of the PCR-positive samples were tested for *Leptospira*-specific IgM; 18 (30%) showed positive results and 5 (8%) showed equivocal results.

Patients with leptospirosis diagnosed by using PCR were 6 to 70 years of age (mean 28, SD 13); 74% were male. Patients with leptospirosis were of similar age and sex as patients with dengue (Table 1). Patients with leptospirosis had less education and came from households with lower income than patients with dengue. The peak occurrence of leptospirosis was in October and November, shortly after the monsoon season in Bangladesh. This overlapped with the period of highest dengue activity (July through December) (Figure).

**Table 1:** Demographic characteristics of dengue patients compared with those of leptospirosis patients, Dhaka, Bangladesh*

Demographic characteristic	Dengue patients (n = 938)	Leptospirosis patients (n = 63)	p value†
Age, y	27.5 ± 11.1	27.9 ± 13.3	NS
Male	694 (74)	46 (73)	NS
Monthly household income (US$)			
<60	174 (19)	20 (32)	0.015
60–119	205 (22)	17 (27)	
120–200	127 (14)	7 (11)	
>200	432 (46)	18 (29)	
Household size	5.4 ± 2.7	4.8 ± 2.1	NS
Level of education			
Illiterate	83 (9)	16 (25)	< 0.001
Primary	144 (15)	13 (21)	
Secondary	424 (45)	22 (35)	
University	214 (23)	8 (13)	
Other or unknown	73 (8)	4 (6)	

*Data are mean ± SD or no. (%). †By Pearson chi-square test or analysis of variance. NS, not significant.

**Figure:**
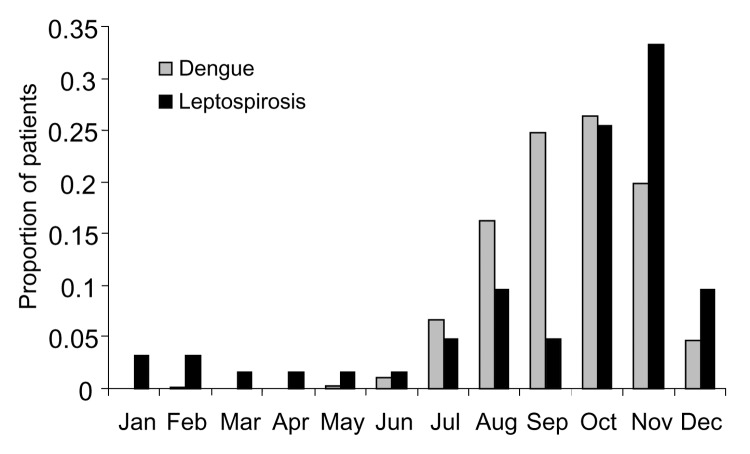
Proportion of dengue and leptospirosis patients at 2 major hospitals in Dhaka, Bangladesh, by month, 2001.

Patients with leptospirosis reported a slightly longer duration of fever than patients with dengue (Table 2). While most patients with dengue or leptospirosis had continuous fever, an intermittent fever was more likely with leptospirosis. Reports of rash were more common with dengue fever. Aside from fever and rash, the symptoms of patients with leptospirosis and dengue were similar (Table 2): headache, myalgia, nausea, and vomiting were most common.

**Table 2:** Clinical characteristics of dengue patients compared with those of leptospirosis patients, Dhaka, Bangladesh*

Clinical characteristic	Dengue patients (n = 938)	Leptospirosis patients (n = 63)	p value†
Symptom			
Duration of fever (days)	5.2 (1–40)	5.8 (1–12)	0.04
Characteristic of fever			
Continuous	894 (95)	53 (85)	0.001
Intermittent	44 (5)	9 (15)	
Rash	562 (60)	24 (39)	0.001
Headache	846 (90)	51 (82)	0.05
Myalgia	836 (89)	53 (85)	NS
Abdominal pain	444 (47)	24 (39)	NS
Pruritus	202 (22)	6 (11)	0.05
Rhinitis	10 (1)	3 (5)	0.01
Nausea	899 (96)	60 (97)	NS
Vomiting	780 (83)	52 (84)	NS
Diarrhea	337 (38)	23 (37)	NS
Physical finding			
Temperature (°F)	98.6 (94–106)	101.4 (98–108)	<0.001
Heart rate	82 (48–160)	90 (60–180)	0.001
Hepatomegaly	79 (8)	7 (11)	NS
Jaundice	17 (2)	3 (5)	NS
Petechial rash	348 (38)	12 (20)	0.005
Positive tourniquet test result (>20 petechiae/inch^2^)	699 (75)	20 (33)	<0.001
Gum bleeding	331 (35)	12 (20)	0.01
Subconjunctival hemorrhage	299 (32)	31 (51)	0.005
Laboratory finding			
Leukocyte count	6.9 ± 5.3	7.0 ± 5.7	NS
% Lymphocytes	47 ± 14	31 ± 15	<0.001
Platelet count (×10^3^/μmL)	85 ± 74	128 ± 83	<0.001
Hematocrit (%)	41 ± 5.8	37 ± 8	<0.001
Outcome of hospitalization			
Recovered or left against medical advice	918 (98.8)	57 (95)	0.048
Death	11 (1.2)	3 (5)	

*Data are median (range), no. (%), or mean ± SD. †By Pearson chi-square test for categorical data and Mann-Whitney U test or analysis of variance for continuous data. NS, not significant.

The median temperature and heart rate at physical examination were higher in leptospirosis patients than in dengue patients. Evidence of bleeding, including petechial rash, positive tourniquet test result, and gum bleeding, were more common in patients with dengue, although they were also found in some patients with leptospirosis. Subconjunctival hemorrhage, which may have been confused with conjunctival inflammation, was more commonly reported in patients with leptospirosis. Hepatomegaly and jaundice were more common in leptospirosis patients, but this difference was not statistically significant.

On laboratory examination, total leukocyte counts were similar in patients with dengue and patients with leptospirosis; however, lymphocytes were more likely to be predominant in patients with dengue. Hemoconcentration and thrombocytopenia were associated with dengue fever.

Of the patients whose outcome was known, 3 (5%) patients with leptospirosis died, compared with 11 (1.2%) patients with dengue (p = 0.048). Antimicrobial therapy for leptospirosis was not provided, and data on the cause of death were not available. The patients who died of leptospirosis were younger than those who died of dengue (24 ± 6 years of age vs. 36 ± 9 years, p = 0.05).

## Conclusions

This is the first description of disease caused by *Leptospira* in urban Bangladesh. Our findings indicate that leptospirosis causes serious febrile illness in the densely populated city of Dhaka.

Studies conducted in other dengue-endemic areas have shown that leptospirosis can be confused with dengue fever (10–12). Most clinical symptoms of leptospirosis patients in Dhaka were nonspecific and not distinguishable from symptoms associated with dengue fever or other viral illnesses. Although fever in leptospirosis patients was higher and of longer duration than in dengue patients, there is sufficient overlap of clinical findings to suggest that clinicians caring for patients in Bangladesh should maintain a high index of suspicion for both diseases, especially during the peak incidence seasons that follow the monsoons. Recognition of leptospirosis is especially important since antimicrobial agents can reduce its severity and duration (13).

Leptospirosis patients identified in Dhaka were impoverished and poorly educated. This may reflect more frequent exposure to environments contaminated with urine from rodents or other animals. In contrast, dengue patients came from households with higher incomes and levels of education. Whether these socioeconomic differences reflect differing patterns of disease can only be determined by future population-based studies, which may in turn shed light on optimal prevention strategies.

Most of the patients hospitalized with leptospirosis and dengue virus infection in Dhaka during the period of this study were male. A recent seroprevalence study in Bangladesh did not demonstrate a sex difference in dengue seropositivity (14), but leptospirosis has been reported predominantly in men in other regions (15). Further prospective research would be useful to better define the clinical spectrum and gender distribution of disease in Bangladesh.

This study has a number of limitations. Only acute-phase serum samples were obtained as part of the hospital-based dengue surveillance program in Bangladesh. Serologic diagnosis of leptospirosis with a single specimen obtained early in infection is limited; hence, we used *Leptospira*-specific PCR for diagnosis in our study population. Although this molecular technique is highly sensitive and specific for the presence of leptospiremia (7), more cases may have been detected through the use of microscopic agglutination testing on paired serum samples. Notably, less than one-third of the patients with a diagnosis of leptospirosis by PCR had detectable levels of *Leptospira*-specific IgM. This is likely due to the sampling of acutely ill patients before seroconversion. In support of this, leptospirosis patients who had detectable levels of IgM exhibited a trend toward longer duration of fever compared with those who did not have detectable levels (6.5 vs. 5.5 days, p = 0.12).

Some cases of leptospirosis resulted in death, and the case-fatality rate among leptospirosis patients was significantly higher than among dengue fever patients. The different case-fatality rates may be related, however, to the intensive training in dengue case management that occurred during this epidemic period, or to the lack of specific antimicrobial therapy for unrecognized cases of leptospirosis. Our findings underscore the need for greater awareness of leptospirosis in the Indian subcontinent, more data on its incidence in Bangladesh, and optimal treatment regimens for leptospirosis that can be applied in resource-poor settings.
